# Supplementary material for: Research on the competitive situation and technical opportunities of global new textile materials technology R&D
Source: PLoS One. 2025 Apr 30;20(4):e0321923. doi: 10.1371/journal.pone.0321923 (PMC12043175; doi:10.1371/journal.pone.0321923)
Supplement: Attachment — Submitted filename: Response to Reviewers.docx [file pone.0321923.s002.docx]

**PLOS ONE Decision: Revision required [PONE-D-24-23639]** –

Dear Editor,

We are truly grateful to yours and other reviewers’ critical comments and thoughtful suggestions on our manuscript (Manuscript ID: PONE-D-24-23639). We have revised the manuscript and responded to these questions point-by-point based on the comments and suggestions. Additionally, the list of changes is given below. Now we are submitting this revised version after making improvements. If you have any other questions about this manuscript, please do not hesitate to contact me.

Reviewer #1: In the manuscript titled “Research on the competitive situation and technical opportunities of global new textile materials technology R&D” the researcher took the 94,525 invention patents for new textile material technology in the world as the research object and explored the competitive situation, technical opportunities, and layout characteristics of technology research and development in this field. .

Here are some points in the current study that need to be addressed for a better understanding of the research.

1. What was the criteria of selecting the patents to be added in this study? And how you have collected the data? Please explain the research methodology in detail.

**Response 1:** Thanks for your suggestions, the data used in this article are from the PatSnap patent database (https:// analytics.zhihuiya.com/). As for new textile material technology, according to the research results of Slubik et al. , Papaspyrides et al. , and Sibiescu et al. , it is mainly defined as high-tech fibers, high-tech fabrics, and high-tech industrial textiles produced by adopting high-tech processes and equipment in recent years. Its content involves textiles, clothing, fibers, spinning, weaving and knitting, etc. The International Patent Classification (IPC) includes two categories: D (textile) and A41 (garment). First you select the new material industry in the national strategic emerging industry classification, and combine it with the international patent classification number D01 fiber, D02 yarn, D03 weaving, D04 knitting, D05 sewing, and D06 fabric treatment, etc.

The process of collecting the data was as follows: firstly, select the patent receiving offices in 170 countries and regions around the world provided by the PatSnap system, and then download the data that meets the requirements according to the criteria. Statistical analysis is carried out by patentometrics to obtain the data of major patentees, technical field and core patents, and finally, the technical opportunities of new textile materials in the world are analyzed through the model.

2. What is meant by “D03 weaving, D04 weaving”, what is the difference between both? Please also explain the term D first then use the 1,2 and 3 etc.

**Response 2:**Thanks for your suggestions, we're so sorry that D03(weaving) and D04(knitting) are not the same, it’s our mistake, we've revised in the manuscript. According to the International Patent Classification(IPC), the technical fields related to patents are divided into eight parts: A, B, C, D, E, F, G, and H. Textile technology, together with papermaking technology, is in the Part D, including D01-D07. D01-D06 represents the aspects of raw materials, spinning, weaving, knitting, sewing and fabric treatment. Please see the official reference URL, https://www.wipo.int/classifications/ipc/en/.

3. The abbreviations are not explained in the text clearly, specifically used in table 4. Please explain.

**Response 3:** Thanks for your suggestions, we have added explanations and descriptions of abbreviations in the manuscript, in particular in Table 4, and highlighted them in different colours.

4. You never mentioned that the patent includes local patents, or these are only international patents?

**Response 4:** Thanks for your suggestions, we selected the patent receiving offices in 170 countries and regions around the world provided by the PatSnap system, and data includes both local patents and international patents.

5. The literature review should be improved. Some other important studies must be added to this research. Below some important studies are referred to enrich the literature review.

• A review of artificial intelligence applications in apparel industry

(DOI: 10.1080/00405000.2021.1880088)

**Response 5:** Thanks for your suggestions, we have enriched the literature review by adding some other important studies, including references to some important studies recommended by reviewers, and the revisions have been highlighted in different colours.

Reviewer #2: The article titled aims to analyze the global landscape of research and development (R&D) in new textile materials technology. It focuses on 94,525 invention patents related to new textile materials, examining the competitive positions, technical opportunities, and R&D layout characteristics. Although the article presents a valuable analysis of the global competitive situation and technical opportunities in new textile materials technology R&D, there are some critical issues and some suggestions for improvement:

• The abstract mentions the analysis of 94,525 invention patents but does not describe the specific methodology used for this analysis

**Response 1:** Thanks for your suggestions, we've added the specific methodology used in the abstract, and the changes have been highlighted in different colors. It reads as follows:

This paper takes the 94,525 invention patents for new textile material technology in the world as the research object,and uses competitive situation calculation and patentometrics to explore the competitive situation, technical opportunities,and layout characteristics of technology R&D (Research and Development) in this field.

• The abstract should succinctly point out the novel aspects of the research, and main contributions to the literature or practice

**Response 2:** Thanks for your suggestions, we've added the novel aspects of the research and main contributions to the literature or practice in the abstract, and the changes have been highlighted in different colors. It reads as follows:

This study effectively supplement the theoretical analysis of competitive situation and technical opportunities of technology R&D, and enrich and expand the empirical research on international new textile materials technology and innovation.

• While the introduction sets the context and significance of the study, it fails to explicitly state the research gap

**Response 3:** Thanks for your suggestions, we've added the research gap in the introduction, and the changes have been highlighted in different colors. It reads as follows:

However, there is a lack of research of the competitive situation and technical opportunities of global new textile materials technology R&D, and the empirical study on international new textile materials technology and innovation can provide a more accurate and comprehensive panoramic analysis, serving a reference for the government's science and technology innovation policy formulation, technology layout and R&D. Therefore, this paper attempts to supplement the existing research in terms of research object and research content, to potentially fill the gap of existing research.

• The introduction does not clearly outline the research methods used in the study. This is a critical component as it provides readers with an understanding of how the research was conducted and ensures transparency. It does not mention similar studies or previous research in the field

**Response 4:** Thanks for your suggestions, we've added the research methods used in the study, and mentioned similar studies or previous research in the field. The changes have been highlighted in different colors. It reads as follows:

Patent measurement is a method to comprehensively understand the level,the new emerging clustering technique and trend of industrial technology development, and technology measurement analysis with patentometrics as the main method is one of the effective and practical methods,including examining the innovation activities of firms in the textile and clothing industry[4-6].

• The introduction should clearly define the research objectives. While the goals are somewhat implied, a concise statement of the specific objectives would enhance clarity. Also, including a clear hypothesis or specific research questions can provide a focused direction for the study and make it easier for readers to understand what the research aims to investigate

**Response 5:** Thanks for your suggestions, we have added specific research questions, and provide a focused direction for the study in the introduction. However, due to the limited space of the manuscript, we're so sorry that we are unable to provide more content.

• The introduction does not provide an overview of the structure of the article. Mentioning the structure helps guide the reader through the paper, making it easier to follow the argument and understand the flow of the research

**Response 6:** Thanks for your suggestions, the structure of the paper is arranged as follows: Literature Review in Section 2, data sources in Section 3, methods in Section 4, results of empirical analysis in Section 5, discussion and conclusions in Section 6, and finally limitations and future research in Section 7.

• The literature review, while well-structured, offers a broad range of research areas but lacks in-depth analysis of existing studies. Specifically, it could benefit from a more rigorous discussion of research on competitive situations, technical opportunities in new material technology, and policy mechanisms for innovation in new textile materials.

**Response 7:** Thanks for your suggestions, however, due to the limited space of the manuscript, we're so sorry that we are unable to provide more content.

• The methodology section is well-defined and detailed, ensuring that the research method is transparent and replicable. However, the abstract and introduction could be improved by briefly mentioning the research method to give readers a more comprehensive overview from the outset.

**Response 8:** Thanks for your suggestions, we have added more content in the abstract and introduction, which give readers a more comprehensive overview from the outset by briefly mentioning the research method.

• To effectively communicate the connection between patent analysis and policy implications, the study should provide a clearer explanation of how the trends of countries and policy recommendations were drawn from the patent data analysis

**Response 9:** Thanks for your suggestions, We have elaborated on the connection between patent analysis and policy implications in the discussion and conclusions of Section 6. However, due to the limited space of the manuscript, we're so sorry that we are unable to provide more content.

• articulate the novel ideas, concepts, or frameworks you introduce to the existing body of knowledge within the field. What are the theoretical contributions of the paper? What are its practical contributions?

**Response 10:** Thanks for your suggestions, we've added the theoretical contributions and practical contributions in the introduction. The changes have been highlighted in different colors. It reads as follows:

The possible marginal contributions of this paper are as follows: (1) Supplementation of the research framework. Based on the 94,525 invention patents for new textile material technology in the world as the research object, this paper constructs the the competitive situation, technical opportunities,and layout characteristics of 170 countries and regions around the world patent technology innovation in order to effectively supplement the analytical framework of international technology R&D. (2) Expansion of research methods. This paper uses competitive situation calculation and patentometrics to scientifically explore the characteristic factors of technology R&D ability and technical opportunities so as to enrich and expand the empirical research on international technology innovation and provide policy enlightenment for optimizing regional sustainable development and national R&D innovation.
