# Supplementary material for: Research on the competitive situation and technical opportunities of global new textile materials technology R&D
Source: PLoS One. 2025 Apr 30;20(4):e0321923. doi: 10.1371/journal.pone.0321923 (PMC12043175; doi:10.1371/journal.pone.0321923)
Supplement: Attachment — Submitted filename: Response to Reviewers2025.docx [file pone.0321923.s003.docx]

**PLOS ONE Decision: Revision required [PONE-D-24-23639]** –

Dear Reviewers

We are truly grateful to yours and other reviewers’ critical comments and thoughtful suggestions on our manuscript (Manuscript ID: PONE-D-24-23639). We have revised the manuscript and responded to these questions point-by-point based on the comments and suggestions. Additionally, the list of changes is given below. Now we are submitting this revised version after making improvements. If you have any other questions about this manuscript, please do not hesitate to contact me.

1. A significant issue that remains unaddressed is the need to communicate the connection between patent analysis and its policy implications. The authors should provide a clearer explanation of how the trends observed in different countries were derived from the patent data analysis, as well as the basis for the policy recommendations suggested. Without a clear understanding of the mechanisms involved, readers will struggle to engage with the results.

**Response 1:** Thanks for your suggestions, drawing on the existing research results, we put the mechanism explanation and the basis for the policy recommendations suggested in the theoretical analysis part of the paper.. It reads as follows:

In recent years, technology competition in emerging industries has become increasingly fierce, and more and more scholars have identified the main competitors in specific technology fields from the perspective of patent competition analysis or patent cooperation analysis, and helped innovation subjects to construct patent strategies and plans to achieve the goal of enhancing competitive advantage by tracking the trend of technology development and evaluating the innovation ability of technology[32].With the rapid development of digital technology, the innovation ability of enterprises has become an important measure of national competitiveness. Therefore, the policy influence of different countries and the research suggestions of scholars have also had a positive effect on the decisive factors for innovative subjects to gain competitive advantages in the market, and provide an important reference for the country to formulate relevant technological strategies and promote the transformation of scientific and technological achievements[33].

At the same time, as the premise of technology R&D and innovation, technology opportunity research has become a key issue of concern to the state and enterprises, and has important strategic significance for technology competition, market layout, regional innovation and industrial development. Therefore, mining technological opportunities from patent data can not only obtain possible new technologies, or new technologies that are improved on the basis of existing technologies, but also provide enterprises with important R&D intelligence and reference directions through analysis[34-36].

2. It is essential that the authors offer a more thorough explanation of the theoretical foundations and their interconnections, even briefly.

**Response 2:**Thanks for your suggestions, we have added a separate section of theoretical analysis to the paper, focusing on explanation of the theoretical foundations and their interconnections. It reads as follows:

# **Theoretical** **analysis**

Patent data mining is the use of patent analysis to extract valuable data information from patent documents, such as the state of industrial technology development, the main areas of technological competition and competitors' technology layout. Through patent competitive intelligence, it helps enterprises understand the industry environment, evaluate themselves and monitor their competitors so as to obtain competitive advantages and potential opportunities[29-31].

In recent years, technology competition in emerging industries has become increasingly fierce, and more and more scholars have identified the main competitors in specific technology fields from the perspective of patent competition analysis or patent cooperation analysis, and helped innovation subjects to construct patent strategies and plans to achieve the goal of enhancing competitive advantage by tracking the trend of technology development and evaluating the innovation ability of technology[32].With the rapid development of digital technology, the innovation ability of enterprises has become an important measure of national competitiveness. Therefore, the policy influence of different countries and the research suggestions of scholars have also had a positive effect on the decisive factors for innovative subjects to gain competitive advantages in the market, and provide an important reference for the country to formulate relevant technological strategies and promote the transformation of scientific and technological achievements[33].

At the same time, as the premise of technology R&D and innovation, technology opportunity research has become a key issue of concern to the state and enterprises, and has important strategic significance for technology competition, market layout, regional innovation and industrial development. Therefore, mining technological opportunities from patent data can not only obtain possible new technologies, or new technologies that are improved on the basis of existing technologies, but also provide enterprises with important R&D intelligence and reference directions through analysis[34-36].

To sum up, the continuous innovation of technology will promote the development of the industry, this process on the one hand requires the progress of technology itself, on the other hand, there is also a demand from the market, the two need to reach a technical research and development in the unity of technical and economic possibilities[37]. Therefore, on the basis of existing research, this paper analyzes the competitive situation and technical opportunities of global textile new material technology research and development in the 21st century, in order to provide a useful reference for the forward-looking and strategic research and development layout of the government, industry and enterprise technology innovation.

3. Additionally, I suggest that the "data sources" section can be placed under the "methods" category for better coherence and organization .

**Response 3:** Thanks for your suggestions, we have placed the "data sources" section under the "methods" category and have moderately adjusted the structure of the paper.
