# Supplementary material for: Research on the competitive situation and technical opportunities of global new textile materials technology R&D
Source: PLoS One. 2025 Apr 30;20(4):e0321923. doi: 10.1371/journal.pone.0321923 (PMC12043175; doi:10.1371/journal.pone.0321923)
Supplement: Attachment — Submitted filename: Response to Reviewers202503.docx [file pone.0321923.s004.docx]

**PLOS ONE Decision: Revision required [PONE-D-24-23639]** –

Dear Reviewers

We are truly grateful to yours and other reviewers’ critical comments and thoughtful suggestions on our manuscript (Manuscript ID: PONE-D-24-23639). We have revised the manuscript and responded to these questions point-by-point based on the comments and suggestions. Additionally, the list of changes is given below. Now we are submitting this revised version after making improvements. If you have any other questions about this manuscript, please do not hesitate to contact me.

Reviewer #1:

1. Break the discussion section into sub-sections with clear headings (e.g., "Regional Innovation Trends," "Challenges in China") for better readability.

**Response 1:** Thanks for your suggestions, we have added the discussion section into sub-sections with clear headings, it reads below:

# . **6. Discussion**

The results of this paper are helpful in sorting out the competitive situation and potential technical opportunities of global textile new material technology R&D. Although the existing research objects in the academic community involve many emerging industries and technologies, there is no research on the global competitive situation and technology opportunity analysis of new textile materials. Therefore, this paper attempts to supplement the existing research in terms of research object and research content, to potentially fill the gap of existing research.

## 6.1 Regional Innovation Trends

The global textile new materials technology innovation is developing rapidly in the direction of high performance, green, intelligence and industrial chain synergy, and around the concept of "revolutionary fibers and textiles", it further integrates interdisciplinary achievements such as nanotechnology and bioengineering to promote the breakthrough of material performance limits. At the same time, digitalization and greening go hand in hand, reshaping the global textile value chain and realizing the transition from "manufacturing" to "intelligent manufacturing". To maintain its global leadership in the field of new materials technology, the United States has set a series of development goals at the comprehensive strategic planning level,including the National Nanotechnology Initiative ( NNI, launched in 2000 ), the Materials Genome Project ( launched in 2011 ), and the National Manufacturing Innovation Network (now known as Manufacturing America) in 2012. It has taken the lead in completing the layout of patented technology research and development and technology opportunity development in the world. Global companies in the chemical industry, advanced materials production, and materials science, represented by Procter & Gamble, Kimberley-Clark Global Ltd., and Dow Global Technologies, provide an important guarantee for the United States to maintain its global leadership position in the development of the new textile materials industry. In addition, Japan's textile new material technology research and development strength is also at the forefront of the world, as early as the 80s and 90s of the last century, the Japanese government began to take a series of measures to promote the development of new material technology, put forward the goal of "focusing on the practicability of new materials, considering the coordinated development of the environment and resources", so it occupies a pivotal international position in the field of cutting-edge materials such as global carbon fiber technology, functional chemical products, new chemical materials, and man-made fibers.

Under the model of full-staff collaboration and innovation that promotes the reform and reorganization of large enterprise groups and encourages the integration of the public and private sectors, South Korea has become an important force in the technical fields of spinning, printing and dyeing, garment production, and textile machinery manufacturing. Since 2012, the government has established policies such as the Nano Convergence 2020 Project, the Third Basic Plan for Science and Technology, and the Korea Future Growth Engines Plan, creating global aramid fiber and new chemical material manufacturers represented by Kolon Industries and LG Chem, which have maintained Korea's global competitiveness in new textile material technology. The development of new textile materials in Europe is similar to that of the United States and Japan, and it attaches great importance to the construction of industrial clusters with its characteristics, establishing and improving the industrial standardization system based on core technology and core manufacturing, and with the intensification of global sustainable development and international trade competition, its new textile material innovation model focuses on industrial demand, emphasizing the added value of textile materials. In 2011, six major technologies, including advanced materials, were identified as key enabling technologies (KETs) for the EU industry, and materials and nanomaterial technology were funded and deployed as important research areas, among which textile new material technology occupies an increasingly important position in the field of EU science and technology development.

## 6.2 Challenges in China

Although China's textile new material technology R&D started relatively late,it has developed relatively rapidly, and has produced several technological achievements that have reached or approached the international advanced level. However, it still faces multiple challenges, mainly involving technology, industry, market and environment. Firstly, in the R&D and application of key technologies and equipment such as new fiber materials, advanced textile products, green manufacturing, and intelligent manufacturing, there is a relatively large gap with developed countries such as the United States and Japan, and the problem of "big but not strong, big but not excellent" is still more prominent, and it is hoped that the country will pay attention to the coherent support and long-term layout of the policy, as well as strengthen international technological innovation and cooperation, encourage domestic enterprises to pay attention to the core patent layout and technological innovation, and actively participate in international competition to enhance the competitiveness of enterprise products and technologies. Secondly, the low conversion rate of achievements of universities and scientific research institutes, the lack of R&D investment of enterprises and other industrial chain coordination and industrialization problems, the dependence on imports of high-end textile equipment and testing instruments, and the pressure of environmental protection and sustainable development restricts the overall upgrading of the whole industrial chain. Finally, the performance evaluation of new materials and the integration of testing standards with international standards need to be further improved, which will affect the market promotion and the competition pattern of application scenarios in emerging fields such as aerospace, defense, and medical.

In summary, with the impact of COVID-19 (Corona Virus Disease 2019) and global geopolitics,countries are paying more and more attention to upgrading supply chain security, so they continue to increase national-level strategic support and investment and begin to promote the reform and restructuring of large enterprises to play a centralized role. Although the United States and Japan still have obvious advantages, the technology gap between various regions is narrowing, and the competition in the field of new textile material technology has entered a white-hot stage.

2. Elaborate on the limitations of the patent database and IP5 scope, explaining how these might affect the comprehensiveness of the findings.

**Response 2:** Thanks for your suggestions, we have added the limitation explaination of the patent database and IP5 scope, which reads as follows:

Although this paper has reference significance and value for the research on the competitive situation and technical opportunities of global new textile materials technology R&D, there are some limitations. Due to limitations of the patent database and IP5 scope, which may not capture all forms of technology R&D collaboration, as some collaborations may not result in patent applications. In the future,more complete global data will be available to compare countries for further and more comprehensive analyses.

3. Compare the findings with existing studies on textile material innovation to position the work within the broader academic discourse .

**Response 3:** Thanks for your suggestions, we have compared the findings with existing studies on textile material innovation, which reads as follows:

4. Revise lengthy and complex sentences to make the manuscript more accessible to a wider audience.

**Response 4:** Thanks for your suggestions, we have revised lengthy and complex sentences to make the manuscript more accessible to a wider audience, all changes are shown in different colors in the paper. .

5. Add a subsection with actionable recommendations tailored to different regions or stakeholders.

**Response 5:** Thanks for your suggestions, we have added a subsection with actionable recommendations tailored to different regions or stakeholders, which reads as follows:

## 6.3 Actionable recommendations

In view of the characteristics of industrial base, resource endowment and policy orientation in different regions, it is suggested that the advantages of each other, and the relevant and feasible R&D strategies for global textile new materials technology are as follows:

(1) In China, promote cooperation between universities and leading enterprises, focusing on the R&D and innovation of high-performance fibers, green materials and degradable textiles. (2) In Japan, we will strengthen the role of Toray, Teijin and other enterprises in technological refinement and high-end material leadership, establish a circular industrial chain of chemical fiber, textile, and consumer brands, and realize sustainable innovation in the circular economy. (3) In South Korea, vigorously develop the application of smart textiles and consumption scenarios, and expand the commercial market of functional materials for international companies such as Samsung Electronics and LG Chem. (4) In the United States, promote disruptive technology incubation and interdisciplinary collaborative development and innovation, promote the application and breakthrough of bioengineering materials in the textile field, and establish a traceability system for textile raw materials based on the blockchain. (5) In Europe, we will continue to strengthen the construction of sustainable standards and recycling systems, promote globally recognized standardization and certification systems, and realize the industrial cluster effect of regional industries such as high-end textiles in Italy, machinery manufacturing in Germany and bio-based materials in Northern Europe.

6. Summarize major findings from the discussion more concisely to avoid repetitive or verbose descriptions.

**Response 6:** Thanks for your suggestions, we have summarized major findings from the discussion more concisely to avoid repetitive or verbose descriptions, all changes are shown in different colors in the paper. .

7. Ensure proper citation of all patent data, government policies, and industrial initiatives mentioned in the manuscript.

**Response 7:** Thanks for your suggestions, we have ensured proper citation of all patent data, government policies, and industrial initiatives mentioned in the manuscript.

8. Emphasize how the findings can inform industry practices, R&D strategies, and policy frameworks.

**Response 8:** Thanks for your suggestions, we have emphasized how the findings can inform industry practices, R&D strategies, and policy frameworks, which reads as follows in the Introduction of paper:

The significance of this study is as follows: (1) Laying a theoretical foundation for future emerging technology cooperation in R&D and innovation. (2) Emphasize the findings can inform industry practices, R&D strategies, and policy frameworks. (3) Through the empirical analysis of global patent data, it provides a reference and basis for the government to rationally allocate innovation resources and formulate relevant policies, which has practical significance and application value for the decision-making of enterprise technology R&D departments.

9. The literature review should be improved. Some other important studies must be added to this research. Below are some important studies that can enrich literature review.

• Comparative Geometrical Analysis of In Situ Mechanical Performance of 2-D Woven In-Plane Auxetic Structures (DOI: 10.1520/JTE20230195)

• Numerical Analysis of Binding Yarn Float Length for 3D Auxetic Structures (DOI: 10.1002/pssb.202000440)

• Influence of tetrahedral architectures on fluid transmission and heat retention behaviors of auxetic weaves (DOI: 10.1016/j.tsep.2023.101946)

• A review of artificial intelligence applications in apparel industry (DOI: 10.1080/00405000.2021.1880088)

• Study of comfort performance of novel car seat design for long drive (DOI: 10.1177/0954407019852279)

• Cellulosic Fillers Extracted from Argyreia Speciose Waste: A Potential Reinforcement for Composites to Enhance Properties (DOI:10.1080/15440478.2020.1856271)

**Response 9:** Thanks for your suggestions, we have improved the literature review, which reads as follows:

# **2. Literature Review**

At present, scholars' research on the technological innovation of new textile materials mainly focuses on three aspects: (1) In the field of textile raw material technology, such as herbal biomass, natural dyes, and extracted cellulose and fiber extraction[7], which are widely used in the development of high-performance, smart textiles, and multi-functional materials field[8, 9], which is to meet the needs of the industry and the market, and improve the added value and competitiveness of textiles. (2) Case studies focusing on technological innovations in new textile materials, such as the use of eco-friendly technologies to produce textiles with the required physicochemical, woven auxetic fabrics and mechanical properties field[10-12],as well as scalable nanofabrication technologies to tailor smart fabrics [13] and natural fabrics [14], to meet various potential application needs. (3) Analysis of the policy mechanism of technological innovation of new textile materials, evaluates the expected impact of new technologies on topics such as the environment, automotive, aerospace, and ballistic areas and human security[15-17], and emphasizes the important role of policy guidance and supervision in the sustainable development of technology. In addition, artificial intelligence (AI) technology is also gaining more and more attention in the apparel industries, and researchers are constantly exploring and optimizing the use of AI techniques in a range of industrial applications[18, 19]. Since patents are synonymous with innovative activities, the analysis of new textile material technology through patents has also attracted the attention of many scholars[20].

Core patents reflect the value of patented technologies, and the corresponding technologies or products can occupy market share and obtain high economic benefits by their competitiveness, so they have a high degree of technological competitive advantage and market economic value[21, 22].At present, academic research focuses more on the technical perspective, pointing out that the core patents are the technical patents that must be used to manufacture a certain technical field or a certain product without circumvention. Some scholars also believe that core patents are patents or patent portfolios with an important economic and strategic value that are in a key position of innovation, influential, and inevitable in a certain technical field[23-25].Based on this research, this paper defines core patents as patents that are in a fundamental position in the technological evolution route,absolutely leading and contributing to the subsequent development of the technology with a high reference value for epitaxial technology.

The research results on technological opportunities were first proposed by Porter et al.[26] in 1995, and its core is to conduct in-depth text mining of academic papers, patent information and network resources, etc.,to identify the interrelationships and development trends of existing technologies, to grasp the latest technological trends, and provide support for future technological innovation of enterprises,which also shows to a certain extent that technology opportunity analysis is essentially the mining of technological knowledge. At present, many scholars have conducted and achieved a series of results on the research in the field of artificial intelligence at different levels from the perspective of scientific literature, involving the knowledge structure, research strength, and future technological trends of expert systems[27-29].As a special scientific document, patents reflect the development of technological innovation as an important output of scientific research and innovation activities[30].Through the mining of patent literature,we can better identify and grasp the potential opportunities in future technology development. In this regard, this paper identifies and analyzes the technical opportunities in the field of new textile materials from three dimensions: innovation subject,patent layout, and technology integration,to provide a useful reference for the government, industry, and research institutions.

In summary, the current research on new textile material technology is mostly aimed at the new textile industry,AI technologies in the apparel industry[31] and the technical field at the level of new textiles, and there are few studies on the competitive situation and technical opportunity analysis of international textile new material technology R&D in academic circles, especially the lack of combining global patent data comparative research. Therefore, this paper takes 94,525 invention patents of new textile material technology in the world as the research object and explores the competition situation, layout characteristics, development trends, and technical opportunities of new textile material technology in major countries and regions in the world,to provide a useful reference for the technological innovation and development of the textile industry.

Reviewer #2:

I appreciate the effort and responsibility you have demonstrated in addressing the previous comments. Compared to the earlier revision, this version reflects a more thoughtful attempt to clarify key aspects of the study.

However, there remains one crucial issue that still needs to be addressed. My expectation was not just a general theoretical discussion on the link between patent analysis and policy recommendations, but rather a clear and explicit explanation of how the specific findings of your study directly lead to the policy recommendations you propose. In other words, while you have articulated the broader theoretical foundation, the manuscript still lacks a concrete connection between the empirical results and the suggested policies in the conclusion.

This connection is essential for ensuring the validity and impact of your conclusions. Without it, the policy implications may appear disconnected from the actual findings, which could raise concerns about the robustness of the study’s contributions. I strongly encourage you to make this link explicit by demonstrating how the trends and patterns observed in your patent analysis serve as the basis for the policy recommendations you suggest.

I look forward to reviewing the revised version and appreciate your efforts in further refining the manuscript.

**Response :** Thanks for your suggestions, we have added the empirical results and the suggested policies in the paper, which reads as follows:

(1) In China, promote cooperation between universities and leading enterprises, focusing on the R&D and innovation of high-performance fibers, green materials and degradable textiles. (2) In Japan, we will strengthen the role of Toray, Teijin and other enterprises in technological refinement and high-end material leadership, establish a circular industrial chain of chemical fiber, textile, and consumer brands, and realize sustainable innovation in the circular economy. (3) In South Korea, vigorously develop the application of smart textiles and consumption scenarios, and expand the commercial market of functional materials for international companies such as Samsung Electronics and LG Chem. (4) In the United States, promote disruptive technology incubation and interdisciplinary collaborative development and innovation, promote the application and breakthrough of bioengineering materials in the textile field, and establish a traceability system for textile raw materials based on the blockchain. (5) In Europe, we will continue to strengthen the construction of sustainable standards and recycling systems, promote globally recognized standardization and certification systems, and realize the industrial cluster effect of regional industries such as high-end textiles in Italy, machinery manufacturing in Germany and bio-based materials in Northern Europe.
